# Galleria mellonella as a consolidated in vivo model hosts: New developments in antibacterial strategies and novel drug testing

**DOI:** 10.1080/21505594.2019.1621649

**Published:** 2019-05-29

**Authors:** Marco Alfio Cutuli, Giulio Petronio Petronio, Franca Vergalito, Irene Magnifico, Laura Pietrangelo, Noemi Venditti, Roberto Di Marco

**Affiliations:** Department of Medicine and Health Sciences “Vincenzo Tiberio”, Università degli Studi del Molise Italy - III Ed Polifunzionale, Campobasso, Italy

**Keywords:** Galleria mellonella, *in vivo* test, antibacterial strategies, novel drug discovery, alternative animal model

## Abstract

A greater ethical conscience, new global rules and a modified perception of ethical consciousness entail a more rigorous control on utilizations of vertebrates for *in vivo* studies.

To cope with this new scenario, numerous alternatives to rodents have been proposed. Among these, the greater wax moth *Galleria mellonella* had a preponderant role, especially in the microbiological field, as demonstrated by the growing number of recent scientific publications. The reasons for its success must be sought in its peculiar characteristics such as the innate immune response mechanisms and the ability to grow at a temperature of 37°C. This review aims to describe the most relevant features of *G. mellonella* in microbiology, highlighting the most recent and relevant research on antibacterial strategies, novel drug tests and toxicological studies. Although solutions for some limitations are required, *G. mellonella* has all the necessary host features to be a consolidated *in vivo* model host.

## Introduction

The use of animal models for scientific purposes dates back to ancient Greece, but since the beginning of the 1900s vertebrate preclinical models have represented the gold standard for *in vivo* tests, as they have provided useful human-like predictions for obtaining mechanistic, efficacious and toxicological information [1]. The fields of drug testing and antimicrobial activity evaluation have been no exception. Here, murine models have often been adopted for infection studies, due to their relatively high similarity to humans about metabolism, body temperature and innate immune response. Nevertheless, these models are expensive and laborious. Moreover, a greater ethical conscience and new global rules and stricter controls mean that it is very time-consuming to obtain authorization for mammalian studies [2,3]. Also, protocols necessitate suitable hosts for the experimental study of *in vivo* infections. Therefore, the selection of alternative models is fundamental for microbiological research, especially when discrepancies in antimicrobial activity are often observed between *in vitro* and *in vivo* testing [4]. Alternatives to rodents have been proposed; the nematode *Caenorhabditis elegans* (*C. elegans*) was the first invertebrate model, followed by adult fruit *larvae* and flies, namely *Drosophila melanogaster* (*D. melanogaster*) [5], and, more recently, *larvae* of the greater wax moth *Galleria mellonella* (*G. mellonella*) [6,7]. This latter organism is widely proposed for the study of pathogenesis, virulence mechanisms, immune response and for the evaluation of the potential of antimicrobial compounds. The use of this mini-host offers economic and ethical advantages compared to mammals and its short lifespan makes it suitable for high throughput studies [8,9].

It may seem futile to expect to obtain clinically useful information from species such as insects and nematodes, but some biological mechanisms, which are very well conserved even in evolutionarily divergent species, share the same evolutionary origins as humans [10]. Indeed, from an evolutionary perspective, insects and vertebrates diverged about 500 million years ago, but many aspects of their physiology remain comparable. Although insects do not possess an adaptive immune response, they own innate immune response mechanisms (at epithelial, cellular and humoral levels) that are surprisingly well preserved and which share part of the evolutionary scale to mammals [11–13]. The lack of adaptive immunity is not a disadvantage, rather the insect models permit the study of host-parasite interactions and related innate-immunity mechanisms without the interference of adaptive responses [14].

A review study conducted by Freires et al. in 2016, showed how the use of alternative models for research purposes had increased dramatically from 1990 to 2015. The most frequently used alternative animal models were: *D. melanogaster* (fruit fly) (41.89%), *Danio rerio* (zebrafish) (29.74%), *C. elegans* (roundworm) (26.53%), *G. mellonella* (greater wax moth) (1.14%) and *Artemia salina* (brine shrimp) (0.70%) [15]. A bibliographic research conducted in March 2019 on PubMed (MEDLINE database), the interest of the scientific community about *G. mellonella* as *in vivo* host model in microbiology has greatly increased, from 2016 to 2018 the scientific articles that have as keywords “*Galleria mellonella*” and “*microbiology*” are more than 42% (292) compared to those published in the last 10 years (691).

Although its physiological characteristics make the *larvae* of *G. mellonella* an ideal host for the study of fungi, especially those of a dimorphic nature [3,16–23], there has been a growth in interest within the scientific community in using the *larvae* for the study of pathogenic bacteria, which has been particularly marked over the last three years. From 2016 to 2018 thirty-seven research papers about new therapeutic strategies for fourteen different bacterial genera were published (Table 1). Moreover, more than thirteen new molecules and four toxicological studies were assessed using the *G. mellonella* model (Table 2).

**Table 1.** *G. mellonella* advantages versus other invertebrate models as *in vivo* model host.

	Invertebrate models			
Characteristics	*G. mellonella*	*C. elegans*	*D. melanogaster*	Advantage
Grown temperature [28]	37°C	From 15 to 25°C	From 16 to 29°C	Mammalian temperature allows temperature-dependent virulence factors studies
Life span [29]	Short [30]	Long [31]	Long [32]	Facilitates experimentation in the laboratory setting
Size (length) [29]	3 to 30mm	1 mm	3 mm	Convenient for handling
Tissue recovery [28]	Possible	Impossible	Impossible	Tissue studies can be performed
Phagocytosis [28]	Present	Absent	Absent	Information about host–pathogen interactions.

**Table 2.** Use of *G. mellonella* in antimicrobial drugs evaluation against pathogens.

Microorganism	Strategy	Reference
Gram-negative		
*Acinetobacter baumannii*	Drug combinations	
	● co-trimoxazole/colistin	[43]
	● levofloxacin/colistin	[44]
	● polymyxin B/netropsin	[45]
	● vancomycin/colistin	[46]
	Drug repurposing	
	● mitomycin C	[47]
	Bacteriophages	
	● *A. baumannii* phage AB-Army1	[48]
	Phytochemicals combination	
	● Theaflavin–Epicatechin	[49]
*Burkholderia spp*	Drug combinations	
	● tobramycin/econazole/miconazole	[4]
	● avibactam/ceftazidime	[50]
	Bacteriophages	
	● *Burkholderia* phage AP3	[51]
	Nutrients from food	
	● fish oils	[52]
	Drug/vitamin combination	
	● vitamin E/norfloxacin	[53]
*Clostridium difficile*	Bacteriophages/drug combination	
	● *C. difficile* bacteriophages lysates cocktail (CDHM1, 2, 5, and 6)/vancomycin.	[54]
*Enterobacter cloacae.*	Drug combinations	
	● imipenem/colistin	[55]
	Bacteriophages	
	● *Escherichia* phage ECP311, *Klebsiella* phage KPP235, and *Enterobacter* phage ELP140	[56]
*Escherichia coli*	Bacteriophages	
	● *Escherichia* phage ECP311, *Klebsiella* phage KPP235, and *Enterobacter* phage ELP140	[56]
*Helicobacter pylori*	Drug repurposing	
	● niclosamide	[57]
*Klebsiella pneumoniae*	Delivery drug system	
	● gentamicin-loaded nanoparticles	[58]
	Bacteriophages	
	● *Escherichia* phage ECP311, *Klebsiella* phage KPP235, and *Enterobacter* phage ELP140	[56]
	● capsule depolymerases produced by the *Klebsiell*a phage KP32	[59]
	● capsule depolymerases produced by the *Klebsiell*a phage KP36	[60]
	● phage ϕBO1E	[61]
*Porphyromonas gingivalis*	Plant extract	
	● *Punica granatum L*.(Pomegranate)	[62]
*Pseudomonas aeruginosa*	Antimicrobial peptide and drug association	
	● Cecropin A2/Tetracycline	[63]
	Antimicrobial peptide	
	● mammalian proline-rich peptide SP-E	[64]
	Quorum sensing targeting	
	● clofoctol	[65]
	Bacteriophages	
	● cocktail of six *P. areuginosa* phages (PYO2, DEV, E215, E217, PAK_P1, and PAK_P4)	[66]
	Proteins isolated from bacteriophages	
	● O-specific polysaccharide lyase from the phage LKA1	[67]
	Plant extract and drug combination	
	● steroidal alkaloids and conessine from *Holarrhena antidysenterica*/levofloxacin	[68]
	Plant made bacteriocins	
	● six pyocins produced by *P. aeruginosa* made using a plant-based transient expression system	[69]
	Nutrients from food	
	● fish oils	[52]
	Drug/vitamin combination	
	● vitamin E/norfloxacin	[53]
*Shigella sonnei*	Phytochemical	
	● geraniol	[70]
**Gram-positive**		
*Enterococcus spp.*	Drug combinations	
	● rifampicin/tigecycline and linezolid/vancomycin	[71]
*Listeria monocytogenes*	Essential oil and phytochemicals	
	● *Cannabis sativa L*. essential oil	[72]
	● trans-cinnamaldehyde, carvacrol, and thymol	[73]
*Mycobacterium abscessus*	Drug combinations	
	● avibactam/piperacillin	[74]
*Staphylococcus aureus*	Drug combinations	
	● pleuromutilins (valnemulin, tiamulin and retapamulin) alone, and in combination with tetracycline or ciprofloxcin	[75]
	Phytochemicals	
	● myricetin	[76]
	● cinnamaldehyde	[77]
	Antibacterial peptide	
	● temporins.	[78]
	Antimicrobial compound	
	● raf-kinase inhibitor GW5074	[79]

As a result, this review aims to highlight the most relevant and most recent research, published from 1 January 2016 to 31 December 2018, on antibacterial strategies, novel drugs testing and toxicological studies conducted with *G. mellonella* as an *in vivo* model.

## In vivo model

The *G. mellonella* insect is a member of the *Galleriinae* subfamily within the *Pyralidae* family of the Lepidopteran order that naturally infests beehives. The greater wax moth develops through four distinct life stages: egg, larva, pupa, and adult. *Galleria larvae* are opaque and white in colour, are about 3 cm long, weigh from 0.3 to 0.5g and undergo a metamorphosis to become grey moths. Temperature is a crucial factor for the development of the insect; the optimum averages are from 29 to 33°C; furthermore, *larvae* can survive at mammalian physiological temperature (37°C) [24,25]. The possibility of breeding *larvae* at a suitable temperature allows experiments to be carried out in conditions that imitate the mammalian body temperature. Indeed several pathogen temperature-dependent virulence factors can be studied using this model [26]. Moreover, temperature plays a key role in pathogen-host interaction, an increase in temperature after bacterial inoculation reduces larval survival [27]. Compared to other invertebrate models, widely used in microbiological research, such as *C. elegans* and *D. melanogaster, G. mellonella* has numerous advantages (Table 3) [28–32]

**Table 3.** *G. mellonella* in novel drugs testing and toxicity screening.

Compound	Antimicrobial target/drug evaluation	Reference
2-aminoimidazole containing urea	*Acinetobacter baumannii*	[82]
1,2,4-triazolidine-3-thiones derivates	*Acinetobacter baumannii*	[83]
Aminoglycoside 6′-N-acetyltransferase type Ib [AAC(6ʹ)-Ib] inhibitory	*Acinetobacter baumannii and Klebsiella pneumoniae*	[84]
1,2-benzisoselenazol-3(2H)-one derivatives	*Enterobacter cloacae*	[85]
Steroid-Au(I)-NHC Complexes	*Escherichia coli*	[86]
[S,S]-ethylenediamine-N,N’-disuccinic acid (EDDS)	*Klebsiella pneumoniae*	[87]
bis-2-aminoimidazole derivative and azithromycin association	*Pseudomonas aeruginosa*	[88]
Silver(I) complex	*Pseudomonas aeruginosa, Escherichia coli, Staphylococcus aureus and Candida albicans*	[89]
Tobramycin−lysine conjugates	*Pseudomonas aeruginosa*	[90]
Hybrid antibiotic tobramycin−moxifloxacin hybrid core structure	*Pseudomonas aeruginosa*	[91]
Marine sponge-derived *Streptomyces* sp. SBT348 extract	*Staphylococcus aureus*	[92]
1-(4-chlorophenyl)-4,4,4-trifluoro-3-hydroxy-2-buten-1-one	*Staphylococcus aureus*	[93]
N-phenyl-1H-pyrazole-4-carboxamide derivatives	*Staphylococcus aureus*	[94]
1,2,3-Triazole/Sulfonate Analogues	Toxicity screening	[95]
Quinoline thiourea compound	Toxicity screening	[96]
Extract from the pulp of *Eugenia brasiliensis Lam. (Myrtaceae)*	Toxicity screening	[97]
Thiazolylhydrazone derivatives	Toxicity screening	[98]

### Immune system

The principal key factor that makes *G. mellonella* a helpful preclinical *in vivo* model is its innate immune response that shares several strategies with the mammalian innate immune system. As mammalians, the innate insect immunity consists of cellular and humoral response and is more advanced than other invertebrates such as nematodes [33]. Of particular interest is the cellular immune response mediated by hemocytes located within the hemolymph. Hemocytes are involved in phagocytosis, nodulation and encapsulation.

The principal mechanisms of pathogen recognition are mediated by:

- hemocytes [6,12,34]
  - recognize pathogenic microorganisms through the direct interaction of their pathogen recognition proteins (PRRs) with pathogen-associated molecular patterns (PAMPs);
  - also indirectly activated by recognition of humoral immune effectors; the Drosophila Toll (like in mammals), and the IL-1 receptor are known to both signal through the NF-KB pathway;
  - once phagocytosed, pathogens are killed by the NADPH oxidase, pathways capable of generating superoxide.
- opsonins [6,35].
  - Apolipophorin-III, peptidoglycan recognition proteins, similar to mammalian opsonins;
  - Hemolin and GmCP8 can recognize and bind to the cell wall of different pathogens including fungi and bacteria and stimulate phagocytosis.

As regards hemocytes, Gongqing et al. in 2016 recognized and characterized four different hemocytes in the hemolymph of the *G. mellonella*, which are known as the plasmatocytes, the granular cells, the spherule cells and the oenocytoids. Plasmatocytes, oenocytoids and above all, granulocytes, have been shown to have the ability to phagocytose bacteria. In addition, a variation in the percentage of hemocytes was observed in the different states of development of the larva. There was also a reduction in the granulocyte count and an increase in the count of oenocytoids during the *G. mellonella* 7th larval instar [36]. These findings led to an important observation: since the variation in the percentage of hemocytes cells changes significantly during the different stages of development of the *larvae*, it is essential for reproducibility that experiments are carried out in the same stage of the larval life cycle. The humoral response of *G. mellonella* includes melanisation catalyzed by phenoloxidase and synthesis of several anti-microbial peptides which play a crucial role in the last line of defence against pathogens [37].

### Antimicrobial peptides (AMPs) and immune-relevant proteins

The humoral response of *G. mellonella* includes a wide range of AMPs: cecropin (active against Gram-positive and Gram-negative bacteria), galiomycin (active against filamentous

fungi and yeast but no antibacterial activity), *Galleria* defensin gallerimycin (active against entomopathogenic fungi but not active against yeast), gloverin and moricins (particularly active against filamentous fungi but also, against yeast, Gram-positive and Gram-negative bacteria) [38]

Together with AMPs, several immune-relevant peptides involved in the immune response of G. mellonella have been identified: Gm proline-rich peptides, Gm anionic peptide, inducible serine protease inhibitor Heliocin-like peptide, lysozyme, moricin-like peptides and x-tox [37–39]. These molecules play a key role in the host defence system, their expression is induced in response to an infection, and it has been observed that their activity is selective towards different pathogenic species. Isolation and characterization of these peptides with antimicrobial activity could be a prospect for the development of novel antimicrobials [34]. Among all the peptides with antimicrobial action, *G. mellonella* produces an insect metalloproteinase inhibitor (IMPI) in response to infection. In 2018, Eisenhardt et al. tested the ability of the fusion protein IMPI-GST (glutathione-S-transferase), produced by fermentation in *Escherichia coli*, to inhibit the proteolytic activity of the M4 metalloproteinases thermolysin and *Pseudomonas* elastase. Results indicated that IMPI is a promising drug candidate for the treatment of *P. aeruginosa* infections [40].

### Influence of diet on larval and immune health

A key factor, often neglected, in experimental infection models is diet. In nature, *G. mellonella* caterpillars feed on honey or other nutrients from the hive (pupa skins, pollen and beeswax) [41] however, when they are raised in the laboratory, a wrong or nutrient-poor diet can cause developmental problems, increased susceptibility to infections or even death [30,42].

Banville et al. have shown the effects of food deficiency on the immune system, a lower density of hemocytes, reduced expression of a range of antimicrobial peptides) and immune proteins have been observed, thus showing a greater susceptibility to *C. albicans* infection [42].

So, to better evaluate a diet suitable for experimental infection models, in addition to the life cycle parameters (duration of the larval stage, weight and percentage of *pupae*), the efficiency of the immune system must also be taken into consideration. For this purpose, Jorjão et al. tested the influence of three different feeding regime on both the larval cycle parameters, together with the volume of the hemolymph and the concentration of hemocytes. The authors showed that a diet that promotes a short larval phase, a weight increase with an enhanced hemolymph volume and a high concentration of hemocytes, also extend larval survival to bacterial (*S. aureus* and *E. coli*) and fungal (*C. albicans*) infections [30].

## G. mellonella as a tool for studying antimicrobial drugs against pathogens

Different therapeutic strategies have been proposed using *G. mellonella* as an *in vivo* model. To overcome antimicrobial resistance and decrease the dosage of individual drugs, a combination of antibacterials has been proposed: drug association, drugs/adjuvant and also drug/antimicrobial peptides. Other effective approaches are the repurposing of drugs that are already in use or even bacteriophage therapy. Moreover, hybrid antibiotics, antimicrobial peptides, delivery drug systems, nutrients from food and vitamins and substances obtained from plants have been evaluated (Table 1).

### Gram-negative

Eleven bacterial species (*Acinetobacter baumanni, Burkholderia cenocepacia, Burkholderia multivorans, Clostridium difficile, Enterobacter cloacae, Escherichia coli, Helicobacter pylori, Klebsiella pneumoniae, Porphyromonas gingivalis, Pseudomonas aeruginosa* and *Shigella sonnei*) belonging to ten different genera are reported in Table 1.

### Acinetobacter baumanni (A. baumanni)

Several drug combinations to treat *A. baumanni* infection have been proposed. A combination of colistin and cotrimoxazole against carbapenem-resistant A. baumannii revealed a greater *in vitro* bactericidal activity and *in vivo* activity than the two drugs alone [43]. Similar results have been obtained with levofloxacin and colistin [44], polymyxin B and netropsin [45], vancomycin and colistin [46] against multidrug-resistant strains. For the treatment of persistent infections, Cruz-Muñiz et al. repurposed the anticancer drug mitomycin C that was able to increase the survival of the insect *larvae* [47].

Regeimbal et al. evaluated the use of bacteriophages as a possible therapeutic strategy and succeeded in attenuating the virulence of a strain of *A. baumanni* pre-exposed to the bacteriophage AB-Army1. In addition to the *G. mellonella* model, other authors have demonstrated the therapeutic efficacy of the mouse full-thickness dorsal infected wound model, confirming the same results [48].

Betts et al. tested two natural polyphenols, theaflavin–epicatechin, alone or in combination against six clinical isolates of multidrug-resistant *A. baumannii. In vivo* results in *G. mellonella* model demonstrated a significantly reduced melanisation score in *larvae* treated with the theaflavin–epicatechin combination compared with monotherapy [49].

### Burkholderia spp

Van den Driessche et al. observed discrepancies in antimicrobial activity between *in vitro* and *in vivo* when tobramycin, econazole and miconazole were tested both in combination and individually. Although *in vitro* tests had demonstrated a synergic effect of the drug combinations, *in vivo* tests demonstrated that neither treatment with tobramycin, miconazole or econazole individually, nor in combination could protect the *larvae* against *B. cenocepacia* infection. The same results were obtained in a mouse pulmonary infection model, corroborating the reliability of *G. mellonella* as a model of infection [4]. Moreover, ceftazidime-avibactam was shown to improve the survival of *larvae* infected with extremely drug-resistant *B. multivorans* isolated from cystic fibrosis patients [50]. Besides antimicrobial drug combinations, the treatment of larvae infected with *B. cenocepacia* isolated from cystic fibrosis patients with *Burkholderia* phage AP3 revealed a significant increase in *larvae* survival in comparison to AP3-untreated infected *larvae* [51].

The antibacterial activity of nutrients from food has also been investigated. Mil‐Homens et al. demonstrated that the survival of *larvae* infected with *B. cenocepacia* and *Pseudomonas aeruginosa* is greater when they are treated with fish oils containing a larger quantity of omega‐3 fatty acids. Furthermore, fish oil has been shown to have a prophylactic effect when given 12 days before bacterial infection [52].

Combinations of drugs and vitamins have also been evaluated using *G. mellonella* as an *in vivo* model. Using D-tocopheryl polyethylene glycol 1000 succinate (TPGS), a water-soluble vitamin E derivative in combination with the bactericidal quinolone norfloxacin, Naguib et al. obtained a significantly increased larval survival rate upon infection. Furthermore, they demonstrated that increased mortality was due to the vitamin E derivative interference with lipocalin binding. Moreover, similar results have been obtained with the same combination against *P. aeruginosa* PAO1 infection [53].

### Clostridium difficile (C. difficile)

Nale et al. assessed *in vivo* colonization of six *C. difficile* clinical strains using single and multiple doses of a filtered *C. difficile* bacteriophages lysates cocktail, both alone and together with vancomycin. The phage cocktail (CDHM1, 2, 5, and 6) was effective as prophylactic therapy and could potentially serve as an intervention therapy to supplement vancomycin and prevent a relapse of the disease [54].

### Enterobacter cloacae (E. cloacae)

A combination of imipenem and colistin against two clinical isolates of *E. cloacae*, led to significantly increased survival of *larvae* when compared to monotherapy alone. The dosages chosen for *in vivo* virulence assay were comparable to those used to treat human infections both for monotherapy and for the two drug combinations [55].

### Escherichia coli (E. coli)

The therapeutic potential of bacteriophages against three multi-drug resistant Gram-negative bacteria was assessed by Manohar et al. The antibacterial proprieties of three different phages, *Escherichia* phage ECP311, *Klebsiella* phage KPP235, and *Enterobacter* phage ELP140, against *E. coli* ec311, *K. pneumoniae kp235* and *E. cloacae* el140, were evaluated in an infection *in vivo* model. Infected *larvae* treated with multiple phage cocktail doses (6h interval) showed a survival rate superimposable to the untreated group (100% survival) with a significant reduction in the count of bacteria in the hemolymph [56].

### Helicobacter pylori (H. pylori)

Tharmalingam et al. repurposed the anthelmintic drug niclosamide and significantly, used it to treat larvae with an *H. pylori* (ATCC 49503) infection and recorded a survival rate of up to 70% compared to the no treatment group [57].

### Klebsiella pneumoniae (K. pneumoniae)

Recently, drug delivery systems as therapeutic strategies have proved to be very promising. Jiang et al. developed and evaluated a poly lactide-co-glycolide/gentamicin formulation capable of successfully treating intracellular *K. pneumoniae* infections. These nanoparticles loaded with gentamicin, have improved larval survival and provided extended prophylactic protection against *K. pneumoniae* [58]. In 2018 Majkowska et al. investigated two capsule depolymerases (KP32gp37 and KP32gp38) produced by the *Klebsiell*a phage KP32 with high specificity for the capsular serotypes K3 and K21, that considerably increased the lifespan of *larvae* infected with *K. pneumoniae* in a time (and strain) dependent manner. Previously, in 2016 the same authors had identified another depolymerase enzyme encoded by the *Klebsiella* phage KP36 (depoKP36) with similar results [59,60]. The activity of the lytic phage ϕBO1E against two carbapenemase-producing *K. pneumoniae* strains (KKBO-1 and KP04C6224) revealed an overall protective capacity for *larvae* from lethal, strain-dependent infection. KKBO-1 was more susceptible to multiple infections than KP04C6224 [61].

### Porphyromonas gingivalis (P. gingivalis)

The glycolic extract of the pomegranate (*Punica granatum L*.) has been assessed for its antimicrobial action. Concentrations that proved to be non-lethal for the l*arvae* showed a high inhibition of the *P. gingivalis* type strain ATCC 33277 inoculated in the *G. mellonella*, in a dose dependent manner. This *in vivo* antibacterial activity may be due to the high presence of gallic tannins and alkaloids in pomegranate extracts [62].

### Pseudomonas aeruginosa (P. aereuginosa)

Antimicrobial peptides, either alone or in combination with conventional antibiotics, have been used successfully to treat *P. areuginosa* infections. Zheng et al. evaluated the efficacy of cecropin A2; a 36-residue α-helical cationic peptide derived from *Aedes aegypti* cecropin A, alone and in combination with tetracycline, against *P. aeruginosa*. The administration of cecropin A2 alone protected the *larvae* and prolonged their survival in a dose-dependent manner. Administration of peptide/drug combinations ensured the survival of all *larvae* tested for more than 96h, demonstrating synergistic protection *in vivo* [63]. An evaluation of the therapeutic activity of proline-rich antimicrobial peptide SP-E, isolated from pig saliva, used against *P. aeruginosa* ATCC 9027 led to a significant increase in the survival of *larvae* in comparison to the untreated group at 6 days post-infection. At this point, 14 of the 16 *larvae* in the control group were dead, whereas eight of the peptide-treated group were still alive [64].

The quorum sensing system, which controls virulence factor production and biofilm formation in diverse human pathogens, is another ideal target for antibacterial therapy. D’Angelo et al. tested the anti-virulence activity of clofoctol, an antibacterial compound that inhibits the expression of the transcriptional regulator PqsR, which control virulence traits in *P. aeruginosa*. Experiments conducted on *P. aeruginosa* PAO1 showed the non-toxicity of the drug at the concentrations used (5mM). Furthermore, the treatment with clofoctol led to a *larvae* survival percentage similar to that observed with the ∆pqsR mutant. Overall, these data demonstrate that clofoctol attenuates *P. aeruginosa* PAO1 lethality in *G. mellonella* [65].

A cocktail of six *P. areuginosa* phages (PYO2, DEV, E215, E217, PAK_P1, and PAK_P4) isolated from strains of different origins has also been tested against P. aeruginosa PAK-lumi both in *G. mellonella* and female BALB/c mice obtaining comparable results. In mice, the cocktail produced a rapid reduction of the bacterial load. In contrast, while using the systemic *G. mellonella* infection model, a significant reduction in the time taken for the infected *larvae* to die (20h) compared to those that were untreated was observed upon phage injection. Moreover, the phage cocktail was able to prevent infection [66].

The effect of a tail spike protein (LKA1gp49) encoded by *Pseudomonas* phage LKA1 on *P. aeruginosa* PAO1 strain pathogenicity has also been evaluated in the *G. mellonella* model. Whether by administering the protein after larval infection or by incubating it with the bacterium an hour before infection, a greater survival rate than the untreated groups was observed during the 72h infection period [67].

Siriyog et al. investigated the efficacy of combinations of steroidal alkaloids and conessine obtained from the Thai medicinal plant Holarrhena antidysenterica with levofloxacin against *P. aeruginosa* strains overexpressing either the MexAB-OprM or MexEF-OprN efflux pumps. Appropriate non-toxic concentrations were determined by injecting *larvae* with triple doses (of the steroidal alkaloids or conessine) for 96h. Combination therapies of conessine or steroidal alkaloids with levofloxacin restored antibiotic efficacy *in vivo*. The enhanced efficacy of the combination treatments was most pronounced with conessine and correlated with a reduced larval burden for those infected with *P. aeruginosa* [68].

Using a plant-based transient expression system, PasÏkevičius et al., were able to produce, isolate and purify six different antibacterial proteins, pyocins, produced by *P. aeruginosa* (S5, PaeM, L1, L2, L3 and a new one, PaeM4). Their antibacterial proprieties against *P. aeruginosa* strains PAO1 and A19 were evaluated with the *G. mellonella* infection model. The survival rates of *larvae* treated with pyocins, especially when mixed together, were significantly higher than those of the untreated group, although the authors observed differences between the two tested strains of *P. aeruginosa*, due to the virulence of different strains [69].

### Shigella sonnei

Geraniol, a natural substance present in the essential oils of plants such as rose and lemongrass, significantly improved larval survival when compared to those that were untreated. Moreover, in a 5-day cytotoxicity experiment, the tolerance level was at least 10-fold higher than the therapeutic level required to control *S. sonnei* infection [70].

### Gram-positive

Five bacterial species (*Enterococcus faecalis, Enterococcus faecium, Listeria monocytogenes, Mycobacterium abscessus* and *Staphylococcus aureus*) belonging to four different genera are reported in Table 1.

### Enterococcus spp

The effects of different antibacterial combinations (rifampicin, tigecycline, linezolid or vancomycin) against five *Enterococcus faecalis* and three *Enterococcus faecium* isolates using an *in vitro* approach and an *in vivo G. mellonella* infection model has been assessed. Antibacterial combinations that displayed synergy in *in vitro* tests were selected for an *in vivo* infection model. Combination treatment demonstrated higher protection of *larvae* post-infection in comparison with antibiotic monotherapy. In particular, rifampicin in combination with tigecycline or vancomycin significantly enhanced survival [71].

### Listeria monocytogenes (L. monocytogenes)

The ability of a sublethal concentration (256 μg/mL) of *Cannabis sativa L*. essential oil extracted from the French monoecious variety Futura 75 to attenuate the virulence of eleven *L. monocytogenes* strains isolated from patients diagnosed with invasive listeriosis has been evaluated by a survival experiment. After 6 days of infection survival rates of infected groups compared with those who were not infected increased remarkably (from 50 to 90%) [72].

The efficacy of three phytochemicals (trans-cinnamaldehyde, carvacrol and thymol), alone and in combination, in reducing the virulence of three strains of *L. monocytogenes*, and their effects on the transcription of antimicrobial peptide genes in *G. mellonella* (responsible for host defence) has also been investigated. In a 5-day infection experiment, all phytochemicals enhanced survival rates. In particular, a combination of carvacrol and thymol was found to be the most effective treatment. Moreover, all phytochemicals were found to upregulate the expression of antimicrobial peptide genes in *larvae* challenged with L. monocytogenes. However, the expression of antimicrobial peptide genes was not significantly affected in uninfected *larvae* treated with phytochemicals [73].

### Mycobacterium abscessus (M. abscessus)

The effect of drug combinations used against the non-tuberculous mycobacterium *M. abscessus* has been evaluated by an *in vivo* experiment. Infection was carried out using the luminescent strain *M. abscessus* mDB158, meropenem, piperacillin and ampicillin alone or with the addition of avibactam in two daily doses during a 72h infection period. *Larvae* treated with either meropenem or piperacillin/avibactam had a significantly lower infection burden compared to the untreated control groups. Piperacillin and avibactam alone had no significant inhibitory effect, similar results were obtained in a single dose experiment [74].

### Staphylococcus aureus (S.aureus)

The *in vivo* antibacterial propriety of three different pleuromutilins (valnemulin, tiamulin and retapamulin) alone, and in combination with tetracycline or ciprofloxacin against two standard *S. aureus* strains (MSSA ATCC 29213 and MRSA ATCC 43300) and two *S. aureus* clinical strains (MSSA N54 and MRSA N9) has been assessed. Administration of pleuromutilins alone was found to lead to increased larval survival, although all combinations with tetracycline provided better results. Among all combinations, tetracycline and valnemulin was the best combination. The combinations with ciprofloxacin did not improve the *larvae* survival rates as compared with monotherapy [75].

The potential protective effect of flavonoid myricetin against *S. aureus* Newman and ATCC 6538 infection in *G. mellonella* has also been investigated. Myricetin was shown to exhibit anti-virulence effects without modulating bacterial growth; indeed, after infection, the treated *larvae* demonstrated increased survival, and larval bacterial counts of the treated group remained similar to those in the untreated control group (PBS-treated) [76].

Another phytochemical, namely cinnamaldehyde, the predominant active compound found in cinnamon oil from the stem bark of the *Cinnamomum cassia*, was found not to induce any toxicity and enhanced the survival rate of *larvae* when they when infected by *S. aureus ATCC 25923*. Moreover, cinnamaldehyde significantly reduced the number of bacteria in *larvae* hemolymph in comparison with that in those in the untreated groups [77].

Mishra et al. demonstrated the efficacy of the antimicrobial peptide Temporin-1OLa (T1OLa), isolated from skin secretions of the *Rana okaloosae* against the methicillin-resistant S. aureus USA300. They found that the deaths of *larvae* were reduced with a single dose of treatment [78].

Johnston et al. tested the ability of a commercially available Raf-kinase inhibitor, namely GW5074 (benzylidene-1H-indol-2-one), to protect *G. mellonella* and *C. elegans* from the methicillin-resistant S. aureus MW2 infection. The administration of a single dose of the compound showed an increase in the survival of species. Moreover, after 5 days of infection about 42% of the G. mellonella *larvae* had survived, demonstrating the long-term protection capacity of GW5074 [79].

## Novel drugs

The excessive and uncontrolled use of antibiotics leads to the spread of bacterial resistance. Several nosocomial and community microbial agents have become resistant to most antibiotics, drastically complicating therapy [80,81]. Therefore, the research for new therapeutic strategies is becoming a pressing issue. As described previously, the *G. mellonella* model represents an important tool for the preliminary screening of antimicrobial compounds. It can be used as an *in vivo* model for a rapid and reliable evaluation of the activity and toxicity of novel antimicrobial drugs and thus should reduce the number of experiments needed using mammalian models.

### Antimicrobial testing

Minrovic et al., carried out a toxicity and antibacterial assay using the *G. mellonella* model to test, *in vivo*, five colistin adjuvants based on a new urea-containing class of 2-aminoimidazole compounds against a highly virulent isolate of *Acinetobacter baumannii* AB5075. During the 24h observation period, an increase in the survival of infected *larvae* treated with five different combinations of colistin and adjuvant compounds was observed [82].

The antibacterial activity of several compounds with 1,2,4-triazolidine-3-thione scaffold against MDR *A. baumanni* AB5075 was assessed by Huggins et al. Although many derivatives had good *in vitro* test results, single dose *in vivo* tests showed conflicting results. When the two most active lead compounds *in vitro* were tested in the *G. mellonella* infection model with AB5075, one drug did not confirm any activity while the other showed only modest activity, partially protecting the *larvae* against infection [83].

Using mixture-based combinatorial libraries, the scaffold ranking approach, and the positional scanning strategy, Tran et al. were able to identify three new aminoglycoside 6′-N-acetyltransferase type Ib (AAC(6ʹ)-Ib) inhibitors, a type of enzyme that induces resistance to aminoglycosides, including amikacin. These molecules, in combination with amikacin, showed good *in vitro* activity (Checkerboard Assay), but further *in vivo* tests demonstrated that only one combination could protect the *larvae* from infection by the resistant strains *A. baumannii A155 and K. pneumoniae JHCK1* [84].

Evaluation of a newly synthesized molecule, derived from the scaffold of Ebslen (an NDM-I inhibitor) as a meropenem adjuvant was assessed by Jin et al. After estimating the non-toxic adjuvant dosage, the *larvae* were inoculated with a lethal dose of carbapeneme-resistant *Enterobacter cloacae* and treated with a combination of meropenem and a new compound or the two alone. Results showed a significantly reduced mortality rate when *larvae* were treated with the drug/adjuvant combination, demonstrating the synergistic action of the two compounds [85].

Velle et al. designed an Au(I)-N-heterocyclic carbene complex bound with either ethynyl oestradiol or ethisterone. Both carbene precursors and complexes containing the oestradiol had antibacterial activity *in vitro* against *E.coli* and *S. aureus*, but *in vivo* tests with *G. mellonella* showed conflicting results. All the complexes tested showed no toxicity and the *larvae* infected with *E. coli* and treated with the compound containing oestradiol demonstrated increased survival, while the carbene precursors did not show any significant increase in larval survival even if *in vitro* studies demonstrated their antibacterial activity [86].

Another molecule which is potentially active against metal-beta-lactamase is zincophore [S,S]-ethylenediamine-N,N0-disuccinic acid (EDDS), which is produced by several bacteria. This zinc chelator has been tested alone and in combination with imipenem against NMD-I producing K. pneumoniae. Treatment with the drugs individually did not result in a significantly higher survival rate for larvae when compared to those left untreated. However, the combination of EDDS and imipenem was proven to be very effective by counteracting the resistance mechanisms of the bacterium [87].

A new therapeutic strategy against *P. aeruginosa* infections conceived by Hubble et al. involved the association of a macrolide (azithromycin) with a new synthetic adjuvant (a bis-2-aminoimidazole derivative) against a highly resistant macrolide, P. aeruginosa laboratory strain PAO1. Authors performed both toxicity and infection studies, demonstrating that the adjuvant is not toxic at the tested concentration and that it increased the survival of *larvae* when administered in a single dose in combination with azithromycin. This is equivalent to the treatment of infected worms with clavulanic acid/penicillin, one of the few clinically approved antibiotic/adjuvant combinations [88].

Jakobsen et al. synthesized and characterized the *in vitro* and *in vivo* anti germicide proprieties of tetrameric-iodine and polymeric silver complexes of the omeprazole scaffold. The tetrameric iodide complex did not show significant antimicrobial action *in vitro* while the polymeric complex showed activity comparable to AgNO_3_. The *in vivo* performance of the *in vitro* active polymeric compound showed less toxicity than AgNO_3_. Moreover, it was able to both significantly stimulate the immune system (increase in blood cell density), and increase the *larvae* survival rate after *Candida albicans, Escherichia coli, Staphylococcus aureus* and *Pseudomonas aeruginosa* infections [89].

A study performed by Lyu et al. reported on the antibacterial properties of new amphiphilic tobramycin-lysine conjugates alone and in association with minocycline and rifampicin. After *in vitro* studies, tests with the *G. mellonella* model were conducted in order to evaluate toxicity and antibacterial action. The *larvae* were infected with XDR *P. aeruginosa* P262, and treated with different non-toxic doses of the drugs, individually or in combination. In the groups treated with minocycline and rifampicin a mortality rate of 100% was recorded, while the combination of these two antibiotics with amphiphilic tobramycin-lysine protected larvae by reducing mortality to 77%. Surprisingly, a lower dose of rifampicin with amphiphilic tobramycin-lysine showed an improved antibacterial action *in vivo* (about 40% survival). This outcome does not reflect the evidence obtained with the *in vitro* tests where the adjuvated minocycline had a higher bacteriostatic activity [90].

Gorityala et al. developed a novel antipseudomonal agent, a tobramycin−moxifloxacin hybrid core structure that protects *G. mellonella larvae* from the lethal effects of MDR *P. aeruginosa* 104354 (resistant to all classes of antipseudomonal agent except colistin). In this compound, moxifloxacin is linked via a C12-tether to the C-5 position of tobramycin. No toxic effects were reported, and efficacy studies of single dose treatment resulted in a 100% survival rate after 24 h, with enhanced long-term survival effects [91].

Balasubramanian et al., found a new bioactive compound (SKC3) isolated from Streptomyces marine sponges derived from *Streptomyces* sp. SBT348. At the concentrations tested the compound showed no *larvae* toxicity in 24h. The antibacterial action observed *in vitro* against *S. epidermidis* RP62A was not confirmed in the *in vivo* model G. mellonella. Indeed SKC3 had not protected the *larvae* from an infection of *S. aureus* USA300 Lac (community-acquired MRSA isolated from a wrist cancer) [92].

A novel protonophore 1-(4-chlorophenyl)-4,4,4-trifluoro-3-hydroxy-2-buten-1-one active versus methicillin-resistant *Staphylococcus aureus* MW2 (MRSA) was tested in *C. elegans* and in *G. mellonella*. The molecule had an antibacterial effect comparable to that of vancomycin in the two models tested, both before and after inoculation of MRSA. The good correlation between the results obtained with two different unconventional models is interesting [93].

The ability of several N-phenyl-1H-pyrazole-4-carboxamide derivatives and other pyrazoles, to improve the survival of wax moth larva infected with S. aureus ATCC 29213 was investigated by Cascioferro et al. After a preliminary *in vitro* screening, the most active compound showed good results after 24h of infection, proving there is a good protective effect. Low toxicity was also demonstrated since there was a slightly lower survival percentage difference between uninfected larvae and those treated [94].

### In vivo toxicity screening

The *in vivo* toxicity assay using *G. mellonella* was performed on two triazole analogues (one derived from carvacrol and the other derived from 2-hydroxy 1,4-naphthoquinone) bearing carboxylic acid, which had previously been tested for their antibacterial activity *in vitro* [95]. Results showed nontoxic behaviour towards the *larvae* viability. Moreover, the hemocyte density of *larvae* treated with tested compounds was not significantly affected if compared to untreated ones [95].

A series of new thiourea-containing compounds, with *in vitro* bacteriostatic activity towards different gram-positive and gram-negative strains, were injected into *larvae* at different doses to determine whether treatment could interfere with normal larval development. The compounds tested were non-toxic because a 100% survival rate was observed in most cases and the number of pupated l*arvae* was comparable to the untreated group [96].

Lazarini et all, tested on *G. mellonella*, an extract from the pulp of *Eugenia brasiliensis Lam. (Myrtaceae)* with a high total phenolic content (389.88 ± 3.48 mg GAE/g). Although this extract showed *in vitro* antibacterial activity (MIC and MBC) against *Staphylococcus aureus* ATCC 25923 (MSSA), *Staphylococcus aureus* ATCC 33591 (MRSA), *Pseudomonas aeruginosa*

ATCC 27853, *Streptococcus mutans* ATCC 700610, *Escherichia coli* ATCC 43895 and *Lactobacillus acidophilus* ATCC 4356 together with an *in vitro* effect on mature biofilm survival, it did not exert toxic effects on the *larvae* when administered at antibiofilm concentrations (10 × MIC −625 μg/mL, which corresponds to doses of 0.025 g/kg of extract). Moreover, the authors did not find the lethal doses able to kill 50% (LD_50_) of the *larvae* [97].

Cruz et al. evaluated the toxicity of thiazolylhydrazone derivatives in *G. mellonella*. Although these compounds showed antifungal activity, the authors observed an LD_50_ < 10mg/kg. This low toxicity was confirmed by a hemolysis assay performed using human erythrocytes [98].

## Limitation

A limitation in the use of *G. mellonella* could be due to its feeding in nature. As reported by Betts et al., the high tolerance of *larvae* to phenolic compounds such as theaflavins and epicatechins could be due to natural adaptation, since bee honeycomb is naturally rich in natural phenolic compounds [49]. These findings are supported by Lazarini et al. testing a plant extract (*Eugenia brasiliensis Lam.)* with a high phenol content. The authors failed to find an LD_50_ using *G. mellonella* as *in vivo* model [97].

Moreover, unlike *C. elegans* and *D. melanogaster*, the *G. mellonella* genome (GenBank NTHM00000000) is still a shotgun project that has not been fully analysed [99]. Also, few microarrays [100,101], libraries of RNA interference and mutant strains are available [6,102]. Recently, the analysis of host-pathogenic interactions at the molecular level using the *G. mellonella* model has shown many advances. Indeed, genome sequencing and studies on the immune response at the proteomic, epigenetic and transcriptomic levels have opened up important new areas of research [100,101,103,104].

As already highlighted by Champion et al. in 2018 [18], the major problem using *G. mellonella* as a non-mammalian infection model is the lack of standardized procedures. Although these authors examined the problem in the field of fungal pathogens, their conclusions can also be extended to bacterial infections. Usually, in most experiments using *larvae* of *G. mellonella*, infection is induced by an intra hemocoelic injection of a titrated bacterial *inoculum* through the last left pro-leg. The response to infection can be assessed by different parameters: mortality, melanization, *larvae* mobility, cocoon formation, quantification of hemocytes and microorganisms in the hemolymph, alteration in gene expression and variations in the proteome [105–107]. Although the infection method and the scoring parameters used in microbial infection studies are very similar, there are numerous experimental variations: preparation or quantity of inoculum injected, source and management of *larvae*, experimental conditions or subjective interpretation of scoring parameters (morbidity or mortality). These diversities can lead to variability in results, which does not allow a direct comparison of different published studies.

Lack of standardization does not only concern the procedures but also the *larvae* of *G. mellonella* used for scientific purposes. *Larvae* can easily be purchased from different commercial sources, as food for reptiles and birds in captivity or as fishing bait and also can be created and standardized directly in research labs [30]. Factors such as age, weight, nutrition and breeding conditions must be known to be able to use these *larvae* as a model of infection, as well as the presence of antibiotics and hormones that can alter their metabolism, generating inconsistent experimental responses [18]. For over a decade, standardized *G. mellonella larvae* have been available on the market, although the cost is higher than that of common *larvae*, these ones offer greater consistency and reproducibility for experiments with pathogenic bacteria [108,109].

## Conclusions

There is undoubtedly a current and increasing interest within the scientific community in using the *G. mellonella* as an *in vivo* pre-clinical model. Although used as a pre-screening procedure, *in vitro* tests alone are often inconclusive, especially in the microbiological field, where new therapeutic strategies or novel antibacterial drugs are sought. Discrepancies in antimicrobial activity between *in vitro* and *in vivo* experiments are often observed. For example, associations of already known drugs [4], probiotic function in humans [110], and even novel synthetic molecules with good *in vitro* activity can have the same or a reduced level of effectiveness or they may not be effective at all when tested with *G. mellonella in vivo* models [83,84,86,90,92]. This may be because common *in vitro* models for susceptibility testing (MIC determination methods and checkerboard assay) involve contact between the bacterium and the drug in a suitable medium, lacking numerous factors present in the *in vivo* models such as the cellular and humoral immunity of the host.

Besides the numerous advantages previously described, *G. mellonella* proves to be very reliable even when compared to other invertebrate models [28,29], as demonstrated in two studies of *S. aureus* methicillin resistance, where the survival rate of the larger wax *larvae* was comparable to that of the nematode *C. elegans* [79,93]. Furthermore, despite the large evolutionary differences between insects and mammals, the results obtained by *G. mellonella* are comparable to those obtained with mice [4,48,66,111,112].

The use of this larval model has provided several advantages over the use of the murine model, including the ability to test many bacterial strains in a limited period at low cost with easy insect management. These characteristics make *G. mellonella* an ideal model for the *in vivo* evaluation of new therapeutic strategies, the efficacy of novel drugs and the characterization of host-pathogen interactions. We strongly believe that, although the use of this insect as an *in vivo* pre-clinical model is now a well-established part of the laboratory routine, the full potential of *G. mellonella* has not yet been developed. Indeed, overcoming some limitations such as the need for full genomic characterization and the formulation of standardized methods are necessary for the progress of scientific research.
